# A pilot comparative study of intraoperative results and surgical outcomes between asleep-awake-asleep and general anesthesia modalities in temporal lobe resections

**DOI:** 10.1007/s10143-025-04112-w

**Published:** 2026-02-05

**Authors:** Daniel San-Juan, Roberto Diaz-Peregrino, Alfonso Arellano-Reynoso, Mario Alonso-Vanegas, Alma Edith Gress-Mendoza, Javier Nieto-Rizo, Erika Aguilar-Castañeda, Miguel Angel Morales-Morales, David Omar López-Hernández, Evelin Zulema Camacho-Castillo

**Affiliations:** 1 Epilepsy Clinic, National Institute of Neurology and Neurosurgery, Mexico City, Mexico; 2 Pedregal Angeles Hospital, Epilepsy Clinic, Mexico City, Mexico; 3 Department of Neurosurgery, University Hospital Heidelberg, Ruprecht- Karls-University Heidelberg, Heidelberg, Germany; 4 Department of Neurosurgery, National Institute of Neurology and Neurosurgery, Mexico City, Mexico; 5 International Center for Epilepsy Surgery, HMG-Coyoacán Hospital, Mexico City, Mexico; 6 Department of Neuro-Anesthesiology, Mexican Institute of Social Security, Medical National Center of Specialties Hospital, Siglo XXI, Mexico City, Mexico; 7 Department of Neuro-Anesthesiology, National Institute of Neurology and Neurosurgery, Mexico City, Mexico; 8 Cognition and Behavior Unit, National Institute of Neurology and Neurosurgery, Mexico City, Mexico

**Keywords:** Mesial temporal sclerosis, Temporal lobe epilepsy, Epilepsy surgery, Awake craniotomy, Asleep–awake–asleep anesthesia, Surgical outcomes

## Abstract

To compare intraoperative findings and surgical outcomes in temporal lobe resections in patients with mesial temporal sclerosis in the language dominant hemisphere performed under general (GA) versus asleep-awake-asleep (AAA) anesthesia modalities. Single-center retrospective case-control study involving 31 adults who had clinical/imaging/neurophysiology concordant evidence of mesial temporal lobe epilepsy in the language dominant hemisphere submitted to temporal lobe epilepsy surgery. GA was used in 20 patients and AAA in 11 patients. Presurgical characteristics of the patients, intraoperative hemodynamic and physiological findings or complications and postoperative outcomes including ILAE scale scores at least 1 year of follow-up were analyzed using descriptive statistics and independent t-tests, Fisher’s exact test, and χ² tests to identify differences between the groups. During the surgery, there were no notable differences between the groups in terms of hemodynamic parameters, arterial blood gas measurements, or bleeding. However, the surgery length was longer in AAA group. Postoperative outcomes, including hospital stay duration, complication rates, and follow-up periods, were also comparable without significant differences. Neurological deficits were minimal in both groups, with no statistically significant differences between them. Most patients achieved positive results based on the ILAE classification in both groups, with most experiencing either no seizures or rare, non-disabling seizures. AAA showed comparable results to general anesthesia, with no intraoperative complications or postoperative negative outcomes. However, due to the limited sample size, further evidence is needed to confirm its benefits in epilepsy surgery involving eloquent areas over GA.

## Introduction

Epilepsy affects approximately 1% of the global population [1]. Between 20 and 40% of patients with epilepsy experience drug resistant epilepsy (DRE) [2]. Of the total of patients with DRE, about 50% are considered candidates for resective epilepsy surgery [3]. Among those undergoing surgery, 40–65% experience a significant reduction or seizure freedom, particularly in cases involving mesial temporal lobe epilepsy due to hippocampal sclerosis (MTLE-HS) [4].

Traditionally, general anesthesia (GA) has been the mainstay for epilepsy surgeries, providing the benefits of a stable surgical environment and patient immobility but limiting surveillance of cognitive functions during resection through real-time functional mapping and neurological assessment [5–7]. The awake craniotomy (AC) anesthetic technique is well-established in neurosurgery, particularly for oncological patients, but it is rarely used in epilepsy surgery, with only a limited number of lesional epilepsy cases reported alongside other non-epileptic brain lesions [8–10]. AC provides several benefits over GA in neurosurgery, including improved precision in identifying and preserving critical brain areas, which can lead to better surgical outcomes and fewer neurological deficits. Additionally, AC shortens the hospital stays, and reduces the need for intensive care unit monitoring, potentially shortening or eliminating ICU stays [11–13].

The AC modalities depend on appropriate anesthetic emergence and the patient’s ability to interact during the psychological evaluation. These include monitored anesthesia care (MAC), asleep-awake-asleep (AAA), and asleep-awake (AA) [8]. MAC sedates the patient while they remain conscious or lightly asleep, with close monitoring of vital signs. AAA involves initial anesthesia, awakening for neurological evaluation, and re-sedation to complete the surgery. The AA method involves initial sedation, waking the patient for the rest of the procedure without re-sedation [14, 15]. Although MAC and AAA have been studied in meta-analyses with significant findings on intraoperative seizures, they still carry risks of bias and require further research [16].

This manuscript aims to conduct a comparative analysis of the AAA anesthetic technique versus traditional GA in the context of MTLE-HS surgery. Our primary objective is to determine whether the intraoperative and postsurgical results are comparable between these two techniques. We hypothesize that there will be no significant differences in both group of outcomes between the AAA and GA techniques. Should our hypothesis be confirmed, this could support the utilization of the AAA modality in epilepsy surgeries in eloquent cortical areas.

## Methods

### Patient population and data source

We conducted a retrospective study at the Department of Neurosurgery of the National Institute of Neurology and Neurosurgery in Mexico City, Mexico, focusing on adult patients who underwent microsurgical resection for MTLE-HS ipsilateral to the language-dominant hemisphere between 2018 and 2023. Our study included patients who met the following criteria: [I] underwent a pre-surgical neurological evaluation at our hospital supported by a epilepsy multidisciplinary committee, and was diagnosed with DRE due to MTLE-HS; [II] had an 1.5 or 3.0 Tesla Brain MRI confirming the presence of unilateral MTLE-HS and correlates with interictal and ictal EEG/VEEG focal epileptiform findings; [III] were submitted to neuropsychological tests to determine the language dominance and cognitive status before surgery and the preservation of language and memory after the surgery; [IV] underwent standard automated campimetry test both before and after the surgery; [V] were followed up for one year with neurological assessments after surgery; [VI] had a histopathological report confirming the diagnosis of MTLE-HS according to the International consensus classification of hippocampal sclerosis in temporal lobe epilepsy from the International League Against Epilepsy (ILAE) commission [17]; and [VII] provided signed informed consent for the use of their medical records for research purposes. Clinical documentation, radiological studies, surgical notes, pathological reports, and subsequent records were retrospectively examined.

### Preoperative neuropsychological evaluation

In the presurgical assessment for epilepsy surgery, several neuropsychological tests were employed to thoroughly evaluate cognitive functions, which were reassessed post-surgery. The Cognistat test provided a broad overview of the patient’s cognitive abilities, assessing key domains such as attention, language and memory [18]. The Rey–Osterrieth Complex Figure Test was administered to evaluate visuospatial abilities and memory [19]. The Rey Auditory Verbal Learning Test assessed verbal learning and memory, providing insights into how well the patient can retain and recall information [20]. The Edinburgh Handedness Inventory (EHI) determines the manual laterality and provides an initial indication of hemispheric dominance [21].

To evaluate language function, the Dichotic Listening or DL technique is a reliable method for identifying auditory processing and language dominance. Patients listen to different sounds in each ear simultaneously and report what they hear, enabling us to precisely determine the language dominance through a laterality index [22]. Verbal Fluency Test was also applied to evaluate the ability to evoke as many words as possible from a category in a given time (60 s), this category such as semantic (animals) and phonemic (words beginning *p*). Additionally, the Boston Diagnostic Aphasia Examination (BDAE) was employed to thoroughly assess language skills, helping us identify any aphasia through tasks such as comprehension, repetition, and reading aloud and The Boston Naming Test (BNT) to assess confrontational picture-naming abilities [23].

### Choice of anesthesia and functional intraoperative monitoring

Patients were selected to undergo either AAA or GA based on the likelihood that the resection would involve eloquent cortical areas related to language, as determined by presurgical evaluations. In cases where language areas were at risk, AAA was offered. However, other factors such as the patient’s ability to tolerate prolonged positioning, significant procedural anxiety, or comorbidities affecting anesthetic stability during the awake phase were also considered. In patients undergoing AAA, intraoperative ECoG and functional language mapping were employed as additional tools to optimize resection while preserving eloquent cortex.

### Anesthesia protocol

#### Preoperative evaluation

Once a potential candidate has been identified by the epilepsy committee, the anesthesiologist conducts an interview to identify comorbidities that could pose a high risk for intraoperative complications, such as morbid obesity, obstructive sleep apnea, bronchial hyperreactivity, asthma, allergies, among others. The airway is assessed using predictive scales for difficult airway management such as Mallampati, Patil-Aldreti, Bellhouse-Dore, and associated risk factors like micrognathia, limited mouth opening, and anatomical dental abnormalities [24, 25].

Difficult intubation is a known cause of anesthetic morbidity and mortality; hence, it is critical for the anesthesiologist to anticipate it during the preoperative evaluation, as airway access and management options are restricted by the Mayfield head holder and limitations in head flexion, extension, and lateralization. It is essential to thoroughly explain the anesthetic technique to the patient, alleviate anxiety, address any concerns, and reassure them that a highly trained team will be managing any intraoperative contingencies [26].

#### Monitoring

The standard monitoring system recommended by the American Society of Anesthesiologists includes electrocardiography, non-invasive blood pressure, pulse oximetry, oxygen analyzer, continuous end-tidal carbon dioxide (ETCO_2_) monitoring, and bispectral index (BIS) [27] monitoring to assess the depth of anesthesia [28].

#### Regional scalp block

The first step in the AAA protocol is achieving satisfactory analgesia and anesthesia through a scalp block targeting six key points. These correspond to branches of the trigeminal nerve and the C2–C3 cervical branches bilaterally [29]. This technique supports maintenance analgesia by reducing nociceptive and reflex stimuli, allowing lower intraoperative opioid doses, smoother emergence, and postoperative pain control [30]. All patients received a scalp block with 0.5% ropivacaine, with approximately 2 to 4 ml injected at each site—supratrochlear, supraorbital, zygomaticotemporal, auriculotemporal, lesser occipital, and greater occipital nerves—for a total of 30 ml, providing intraoperative analgesia lasting 6 to 8 h. For procedures lasting longer than 6 h, the scalp block was repeated at the end of surgery to ensure postoperative analgesia [31, 32].

#### Airway management

##### First phase: patient under anesthesia

A difficult airway cart was prepared prior to induction, equipped with a fiberoptic bronchoscope, videolaryngoscope, laryngeal masks, and endotracheal tubes of various sizes [33]. Once the surgical safety checklist was announced, total intravenous anesthesia was initiated using target controlled infusion with Medcaptain HP infusion pumps, administering dexmedetomidine, fentanyl, and propofol [34].

A laryngeal mask was chosen to reduce airway stimulation and coughing reflex during intraoperative awakening. The patient was preoxygenated with O₂ via adult face mask at 3–5 L/min for 3 min. Dexmedetomidine infusion was initiated followed by fentanyl. After 2 min, a bolus of lidocaine was administered to suppress the cough reflex and as an adjunct. After a 3-minute latency, propofol was started with a total latency of approximately 6 min until reaching pharmacologic targets [35, 36].

A supreme laryngeal mask size 3, 4, or 5 was then inserted depending on patient weight. The cuff was inflated to maintain 30–35 cm H₂O as measured by manometry. Absence of air leaks was confirmed, and ventilation was adjusted to achieve ETCO₂ of 30–35 mmHg, SpO₂ > 95%, a tidal volume of 6 ml/kg, and BIS between 40 and 50 [37].

Once the airway was secured, the scalp block was performed and cranial fixation with the Mayfield head holder was applied, ensuring alignment of the three airway axes (oral, pharyngeal, and laryngeal) and maintaining anesthesiologist access throughout the surgery. The patient was positioned comfortably for emergence, with the thorax elevated at 30 degrees and other joints at 15 degrees, and all bony prominences protected [25].

At the beginning of the surgical approach, nociceptive stimulation is usually minimal, allowing reduction of anesthetic infusions: dexmedetomidine was decreased and stopped 30 min before awakening [38]. Fentanyl was reduced and discontinued once dural opening (previously infiltrated by the surgical team) was completed. Adjunct medication included paracetamol 1 g, an antiemetic, and a nonsteroidal anti-inflammatory drug [37].

##### Second phase: intraoperative awakening

Once the dura mater was infiltrated and dural opening completed, propofol infusion was stopped. A second dose of lidocaine was administered. BIS monitoring was used to guide awakening; once BIS exceeded 80, verbal stimulation was applied until anesthetic emergence occurred. The laryngeal mask was removed, nasal cannula with capnography was placed at 2 L/min, and sedation level was maintained at Ramsay 2 and RASS 0. After confirming satisfactory awakening, the neuropsychologist was allowed to perform motor, sensory, and language testing [25].

##### Third phase: patient under anesthesia again

After completing intraoperative neuropsychological testing, general intravenous anesthesia was resumed, and the airway was resecured using either a laryngeal mask or orotracheal intubation via videolaryngoscope or fiberoptic bronchoscope. The same induction doses were used to complete the surgical procedure. Alternatively, at the anesthesiologist’s discretion, sedation was deepened with dexmedetomidine restarted to complete the surgery while maintaining spontaneous ventilation and avoiding airway instrumentation [25].

If the surgery lasted more than 8 h, the scalp block was repeated at the end of the procedure and before patient awakening to provide postoperative analgesia, along with a second dose of NSAIDs, acetaminophen, and an antiepileptic drug [38]. For anesthetic drug dosages and infusion parameters, see Table 1.

**Table 1:** Anesthetic Protocol, medication Dosing, and airway maintenance parameters for awake craniotomy

Phase	Medication	Dose/Target	Notes
Under anesthesia	Dexmedetomidine	I. 0.2 ng/ml Cp II. 0.1 ng/ml Cp at the beginning of the surgical approach III. Stopped 30 min before awakening	Hannivoort pharmacokinetic model
	Fentanyl	I. 2–3 ng/ml Ce II. 1.5–1.8 ng/ml Ce at the beginning of the surgical approach III. Stopped after dural opening	Shafer pharmacokinetic model
	Lidocaine	1 mg/kg bolus	For cough reflex suppression
	Propofol	2.5–3 mcg/ml Ce	Eleveld model; BIS 40–50
	NSAID	Standard dose	Analgesic adjunct
	Paracetamol	1 g	Analgesic adjunct
	Antiemetic	Standard dose	postoperative nausea and vomiting prophylaxis
	Airway	Supreme LMA size 3–5	- Cuff 30–35 cm H₂O - ETCO₂ 30–35 mmHg - SpO₂ > 95%, a tidal volume of 6 ml/kg
	Other considerations	—	- Scalp block - Patient positioning
Intraoperative awakening	Propofol	Discontinued	To allow emergence
	Lidocaine (IV)	1 mg/kg bolus	Prior to awakening
	Airway	Remove LMA	Nasal cannula 2 L/min + capnography
	Sedation target	Ramsay 2; RASS 0	BIS > 80 before verbal stimulation
	Testing	—	Motor, sensory, and language testing
Under anesthesia again	Propofol/Fentanyl	Same targets as First Phase	Reinduction after testing
	Dexmedetomidine	0.3 ng/ml Cp (optional)	For deep sedation with spontaneous ventilation
	Airway	LMA or orotracheal tube	Based on anesthesiologist preference
	Analgesia (long procedures)	Repeat scalp block; NSAIDs; acetaminophen	If surgery > 8 h
	Antiepileptic drug	Standard dose	Postoperative seizure prophylaxis

Abbreviations: *BIS*, Bispectral Index; *Ce*, effect-site concentration; *Cp*, Plasma concentration; *ETCO₂*, End-tidal carbon dioxide; *LMA*, Laryngeal Mask Airway; *SpO₂*, peripheral capillary oxygen saturation; *RASS*, Richmond Agitation–Sedation Scale.

### Intraoperative neuropsychological and neurophysiological tests

Only patients in the AAA group underwent cortical (Penfield technique [60 Hz, 4 s]) and subcortical electrical (3.11 Hz continuous stimulation) language mapping using a bipolar probe, ranging from 1 to 7 mA and 0.5 ms, according to standard intraoperative awake electrical mapping protocols using a Ojemann OCS2^®^ (New Jersey, USA) [39]. BDAE and BNT were administered simultaneously, encompassing picture naming, auditory comprehension, and spelling [40]. Cortical testing sites were separated by 1 cm and tested non-sequentially three times each for 3 to 4 s, with a 4 to 10-second inter-task interval. If patients were fatigued or struggled with the testing, the inter-task interval was extended to allow for additional recovery time. A site was considered ‘positive’ if it produced either speech arrest without a simultaneous motor response, anomia or aphasia in two of the three attempts. These positive sites were recorded along with the stimulation parameters and marked with numbered indicators. Subcortical mapping was performed similarly but focused on nearby areas with presumed language function [39, 40]. Motor and sensory functions were evaluated neurologically during the awake phase of the neurosurgery.

### Surgical procedures

The standard ECoG-guided corticoamygdalohippocampectomy (CAH) was performed in both GA and AA Groups. The extent of resection was adjusted according to the epileptogenic zone and based on language mapping results in the AAA group. In the standard CAH, the patient is positioned supine with the head tilted to access the medial temporal structures via a question mark-shaped incision beginning at the zygoma and extending to the hairline. Following a temporalis muscle reflection and a pterional craniotomy, the dura is opened, cortical and subcortical electrical mapping is carried out followed by a basal T3 resection to expose and resect the mesial temporal structures. The resection extends laterally from the temporal pole, varying based on the hemisphere to minimize postoperative language deficits. Key structures, such as the hippocampus and amygdala, are carefully manipulated and completely removed [41].

### Intraoperative neurophysiological monitoring (IONM)

In both patient groups, intraoperative neurophysiological monitoring (IONM) was performed using the Cadwell IOMAX system (Kennewick, WA, USA). An 8, 16 or 20 -contact subdural cortical strip or grids electrodes (Filters: 0.3–70 Hz, 50–100µV/Div) were used for intraoperative electrocorticography (iECoG), initially positioned over the lateral temporal neocortex and subsequently placed within the resection cavity to assess residual epileptiform activity. Pre-resective iECoG was obtained after stabilization of anesthetic depth and physiological parameters, allowing identification and localization of interictal epileptiform discharges (IEDs) such as spikes, sharp waves, polyspikes, and paroxysmal fast activity. These findings were used to confirm concordance with the preoperative epileptogenic hypothesis and to define whether neocortical or hippocampal resection trajectories required adjustment [42, 43].

Post-resection iECoG was then performed to assess whether epileptiform activity persisted at the resection margins. A “negative ECoG” was defined as the absence of IEDs during a 3 to 10-minute recording epoch; isolated sharp transients without evolution were not considered pathological. Resection was considered complete when a negative ECoG was achieved. This operational definition is consistent with criteria used in our center and major epilepsy centers and publications reporting the prognostic value of post-resection ECoG silence [42, 44].

### Postoperative follow-up

All patients were evaluated for at least one year postoperatively for neurological and systemic complications, as well as neurological deficits such as visual field defects and language impairments. Seizure outcomes were assessed using ILAE Classification of Postoperative Outcomes.

### Statistical analysis

Statistical analyses were computed using SPSS software (IBM, version 25.0). To identify statistical differences among anesthesia modalities, we employed the Fisher exact test for binary categorical variables and the χ2 test for categorical variables with more than two levels. The variables examined included sex, EZ found in neuroimaging, language location ipsilateral to the EZ, ASA score, complications during the surgery, language impairment and visual field deficits < 12 months and > 12 months, and the ILAE score outcome classification at last follow-up. Dichotomous and ordinal variables are presented as number and percentage. Independent T-tests were employed to assess continuous variables such as age, monthly seizure frequency, heart rate, mean arterial pressure, systolic pressure, diastolic pressure, pO2, pCO2, surgery duration, bleeding during surgery, days of hospitalization and time of follow-up. Continuous variables were displayed as mean and standard deviations. A p-value < 0.05 was considered statistically significant.

## Results

Thirty-one patients met the inclusion criteria, of whom 42% were women. The mean age of the participants was 36.6 ± 11.3 years. Twenty patients underwent GA and 11 AAA. The mean follow-up duration was 2.7 ± 1.1 years. The language dominance was 100% ipsilateral to MTLE-HS in both groups. No deaths were reported during or after the surgery. None of the patients required reintervention or admission to the ICU.

### Presurgical characteristics of the patients based on the anesthesia modality

The mean age was comparable between groups, with a higher proportion of female patients in the GA group compared to the AAA group, though this difference was not statistically significant. Both groups had comparable ASA scores. Monthly seizure frequency was higher in the GA group, but this difference was also not statistically significant. Only twelve patients from the GA group and seven patients from the AAA group presented with short-term memory loss as a neurological deficit before surgery (Table 2).

**Table 2:** Presurgical characteristics of the patients based on the anesthesia modality

I. Patient characteristics			
Characteristics	GA	AAA	*p* Value
Number of patients	20/31	11/31	
Age in years^‡^	39.5 (9.3)	33.6 (13.3)	0.158
Female sex	9 (45)	4 (36)	0.718
ASA Score			0.052
2	11 (55)	2 (18)	
3	9 (45)	9 (82)	
Seizure frequency per month^‡^	13.6 (20.1)	10.6 (10.8)	0.659
Time from epilepsy diagnosis to last follow-up in years^‡^	32.5 (11.1)	25.9 (9.7)	0.441
Epileptogenic zones in neuroimaging			0.071
Right temporal	3 (15)	2 (18)	
Left temporal	17 (85)	9 (82)	
Neurological deficit			
Memory loss	12 (60)	7 (64)	0.577
Visual field defects	0	0	1.000
Language impairment	0	0	1.000

*Pearson’s chi‑square test and* ^***‡***^independent T-test; *Statistically significant

Values are the number of patients (%); ^‡^Values are means (Standard Deviations)

Abbreviations: *AAA*, Awake-Asleep-Awake; *GA*, General anesthesia.

All patients had concordant imaging and video-EEG data of MTLE-HS epilepsy; 26 cases were left sided. In only 5 cases the right temporal lobe was the surgical target, and the language-dominant hemisphere was ipsilateral, with 3 cases in the GA group and 2 in the AAA group (Table 2).

### Intraoperative findings during epilepsy surgery based on the anesthesia modality

**Fig. 1:**
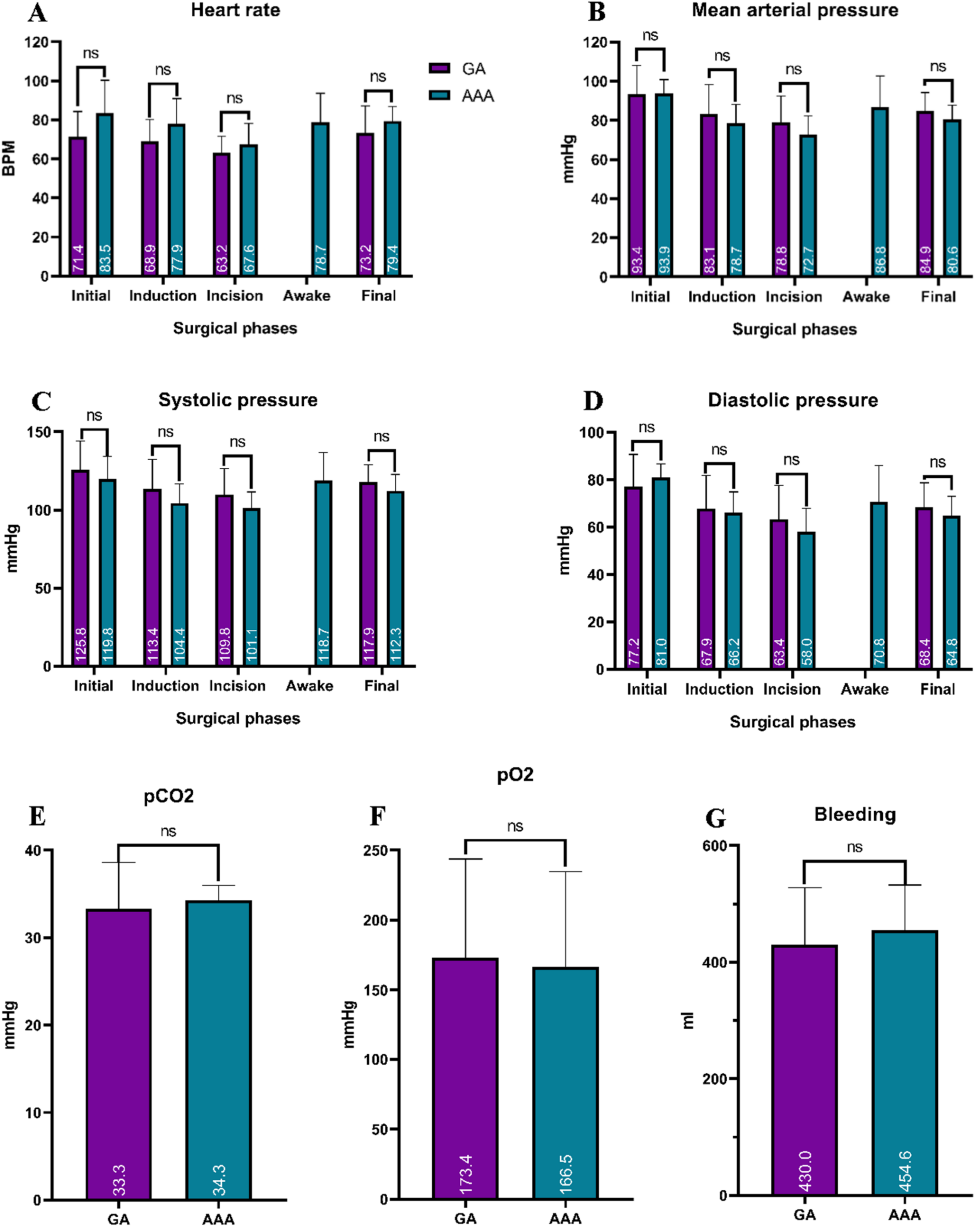
Intraoperative findings during epilepsy surgery based on anesthesia modality. Panels (A–G) display key vital parameters recorded intraoperatively: (**A**) heart rate (beats per minute), (**B**) mean arterial pressure (mmHg), (**C**) systolic blood pressure (mmHg), (**D**) diastolic blood pressure (mmHg), (**E**) arterial pCO₂ (mmHg), (**F**) arterial pO₂ (mmHg), and (**G**) estimated blood loss (mL). Data is grouped according to the type of anesthesia administered: AAA (Asleep-Awake-Asleep) and GA (General Anesthesia)

The analysis of hemodynamic parameters at four key time points—initial, induction, incision, and end of surgery—showed no statistically significant differences between the two groups in terms of heart rate, mean arterial pressure, and systolic and diastolic pressures. Additionally, arterial blood gas values, as well as the intraoperative bleeding, were comparable in both groups without significant differences. Only the surgery duration was longer in the AAA group compared to GA (8.6 ± 1.9 and 7.1 ± 1.3 h; *p* = 0.012). Importantly, during the awake periods, heart rate and blood pressure remained within physiological ranges, further indicating hemodynamic stability during the AAA modality (Table 3 and Fig. 1). No intraoperative seizures were recorded in either group and no neurological or neuropsychological tests were aborted during the AAA approach.

**Table 3:** Intraoperative findings during epilepsy surgery based on the anesthesia modality

II. Intraoperative parameters			
Characteristics	GA	AAA	*p* Value
Number of patients	20/31	11/31	
Heart rate in BPM			
Initial	71.4 (12.9)	83.5 (16.8)	0.054
Induction	68.9 (11.3)	77.9 (13)	0.078
Incision	63.2 (8.5)	67.6 (10.5)	0.241
During the awake period	-	78.7 (14.9)	-
End	73.2 (13.9)	79.4 (7.5)	0.186
Mean arterial pressure in mmHg			
Initial	93.4 (14.6)	93.9 (6.9)	0.914
Induction	83.1 (15.2)	78.7 (9.5)	0.401
Incision	78.8 (13.7)	72.7 (9.6)	0.080
During the awake period	-	86.8 (15.9)	-
End	84.9 (9.3)	80.6 (7.1)	0.188
Systolic pressure in mmHg			
Initial	125.8 (18.2)	119.8 (14.4)	0.356
Induction	113.4 (18.9)	104.4 (12.3)	0.169
Incision	109.8 (16.6)	101.1 (10.4)	0.089
During the awake period	-	118.7 (18.1)	-
End	117.9 (11.1)	112.3 (10.4)	0.169
Diastolic pressure in mmHg			
Initial	77.2 (13.5)	81 (5.8)	0.283
Induction	67.9 (13.9)	66.2 (8.7)	0.723
Incision	63.4 (14.2)	58 (9.9)	0.274
During the awake period	-	70.8 (15.2)	-
End	68.4 (10.3)	64.8 (8.2)	0.329
pO2 in mmHg	173.4 (70.3)	166.5 (68.3)	0.794
pCO2 in mmHg	33.3 (5.3)	34.3 (1.7)	0.445
Surgery duration in hours	7.1 (1.3)	8.6 (1.9)	0.012*
Bleeding during surgery in ml	430 (97.9)	454.6 (77.9)	0.881

Independent T-test; *Statistically significant

Values are means (Standard Deviations)

Abbreviations: *AAA*, Awake-Asleep-Awake; *GA*, General anesthesia

In the AAA group, ECoG was performed in all cases and consistently identified the EZ, and language mapping reliably detected positive language sites in every patient. Postsurgically, a final ECoG confirmed the absence of residual epileptiform activity in all cases. The CAH matched the planned surgery in all AAA cases (Table 4). Similarly, all resections in the GA group strictly adhered to the predefined preoperative CAH margins.

**Table 4:** Intraoperative mapping and resection extent in patients submitted to AAA

Patient	Language dominance	ECoG performed and EZ identified	Language mapping (+)	Resection modified due to mapping	Description of modification	Post-OP language modification
1	Left	Yes	Yes	No	-	No
2	Left	Yes	Yes	No	-	No
3	Right	Yes	Yes	No	-	No
4	Left	Yes	Yes	No	-	No
5	Left	Yes	Yes	No	-	No
6	Left	Yes	Yes	No	-	No
7	Left	Yes	Yes	No	-	No
8	Left	Yes	Yes	No	-	No
9	Left	Yes	Yes	No	-	No
10	Left	Yes	Yes	No	-	No
11	Right	Yes	Yes	No	-	No

Abbreviations: *AAA*, Awake-Asleep-Awake; *ECoG*, Electrocorticography; *EZ*, Epileptogenic zone

### Post-surgical outcomes of the patients based on the anesthesia modality

Hospitalization durations ranged from 5.3 to 7.3 days in both groups, with no significant differences. Complication rates were 0% in the GA group and 9% in the AAA group, corresponding to one patient with a surgical site infection; this difference was not statistically significant. Neurological deficits were documented only in the GA group, affecting 10% of patients within the first 12 months and 5% thereafter, all presenting as visual field impairments in the form of contralateral quadrantanopia, with no significant difference found between the groups. Regarding seizure outcome, 75% of patients in the GA group and 55% in AAA group were seizure free, classified as ILAE Scale Class 1 (Table 5).

**Table 5:** Post-surgical outcomes of the patients based on the anesthesia modality

III. Postsurgical outcomes			
Characteristics	GA	AAA	p Value
Number of patients	20/31	11/31	
Days of hospitalization^‡^	5.3 (1.8)	7.3 (3.9)	0.128
Any complication after surgery	0	1 (9)	0.355
Neurological deficit < 12 months			
Visual field defects	2 (10)	0	0.409
Language impairment	0	0	1.000
Neurological deficit > 12 months			
Visual field defects	1 (5)	0	0.591
Language impairment	0	0	1.000
ILAE outcome classifications at last follow-up			0.064
Class 1	15 (75)	6 (55)	
Class 2	1 (5)	2 (18)	
Class 3	4 (20)	2 (18)	
Class 4	0	1 (9)	
Class 5	0	0	
Class 6	0	0	

Pearson’s chi‑square test and ^***‡***^independent T-test; *Statistically significant

Values are the number of patients (%); ^‡^Values are means (Standard Deviations)

Abbreviations: *AAA*, Awake-Asleep-Awake; *GA*, General anesthesia

## Discussion

Our study confirms our hypothesis that there are no significant differences in intraoperative results or postoperative outcomes between AAA and GA. The presurgical characteristics of patients in the GA and AAA groups were comparable in terms of mean age, ASA scores, and seizure frequency, with the main differences being a different distribution of EZ and language dominance. Intraoperatively, there were no significant differences between the two groups regarding hemodynamic parameters, arterial blood gas values, or bleeding. Only the surgery duration was longer in the AAA group. Postoperatively, hospitalization duration, complication rates, and follow-up periods showed no significant differences. Neurological deficits were observed only in the GA group. Most patients in both groups achieved favorable outcomes according to the ILAE classification, experiencing either no seizures or infrequent, non-disabling seizures.

### Presurgical characteristics of the patients based on the anesthesia modality

The evaluation of candidates for epilepsy surgery includes a comprehensive understanding of a patient’s cognitive and behavioral functions [45, 46]. Neuropsychological evaluations are essential for identifying eloquent areas near the affected region [47]. The central study for language dominance was the DL technique. No additional tests such as Wada or language fMRI were performed to determine language dominance. Based on current clinical criteria [48], Wada testing is typically reserved for cases with atypical or inconclusive language lateralization, bilateral language representation, or bitemporal dysfunction, none of which were present in our cohort. Regarding non-invasive methods, the DL technique included in our protocol has demonstrated high concordance with fMRI [49, 50], supporting its validity as a reliable non-invasive tool for language lateralization. One technical limitation in our patients with suspected right-hemispheric language dominance was that language lateralization was assessed only using DL technique. Although fMRI, TMS, or the Wada test were not used, intraoperative mapping reliably identified positive language sites. However, fMRI and the Wada test will be incorporated into our protocol in cases where language lateralization remains unclear with DL, or when bilateral or right-hemispheric dominance is suspected.

While some research suggests that locating eloquent areas near potential resection sites could lead to worse outcomes [51], awake procedures are benefited for the identification of these areas in epilepsy, improving surgical planning and minimizing postoperative deficits [52]. Hermann and Wyler observed that patients with preserved eloquent areas undergoing temporal lobe epilepsy surgery in the dominant hemisphere exhibited significant gains in receptive language comprehension and associative verbal fluency [53].

The indication for using the awake surgery protocol in our series was language dominance in an eloquent area in cases where resection could be extended to the superior temporal gyrus. A similar strategy was observed in the study conducted by Guibourd de Luzinais and colleagues, where 36.4% of their patients had surgical planning adjusted to preserve parts of the eloquent area [52]. Conversely, the primary reasons for choosing GA with ipsilateral language dominance to the surgical site were a well-defined EZ that did not require further delineation beyond the planned CAH. Additional reasons for not performing AAA included low tolerance for prolonged lying down, significant procedural anxiety, and comorbidities that compromised stable anesthesia during the awake phase [54].

### Intraoperative findings during epilepsy surgery based on the anesthesia modality

The two groups had similar clinical and sociodemographic characteristics, allowing for a fair comparison of surgical outcomes in our work (Table 2). Intraoperatively, both blood pressure and heart rate remained within physiological ranges, with no statistical differences between the GA and AAA groups (Table 3; Fig. 1). Dilmen et al. reported elevated blood pressure in their AAA group during neurological exams, requiring antihypertensive intervention, likely due to a sympathetic response upon patient awakening. The authors linked the elevated blood pressure to an increase in intraoperative bleeding [37]. Rajan et al., on the other hand, observed lower but physiological systolic, diastolic, and mean arterial pressures, as well as heart rates in the AAA group. This was attributed to the use of local anesthesia and dexmedetomidine, which helped suppress sympathetic responses during pinning, excision, and emergence phases of surgery [55]. Although dexmedetomidine represents a higher-cost agent [56], we believe it could be a valuable addition for managing potential elevations in blood pressure and heart rate, as well as alleviating patient anxiety during the awake period. It has been safely utilized as a standard medication in previous AC procedures [34].

In our protocol, hyperoxygenation was crucial in anesthesia (Table 3 and Fig. 1) as it prevents hypoxemia by increasing oxygen saturation prior to induction, extends safe apnea time during intubation, and improves tissue oxygenation. However, careful management is essential due to risks of oxygen toxicity and lung injury from prolonged hyperoxygenation [57–59]. In our case, the benefits of hyperoxygenation outweighed the risks, as it maintained normal PaCO2 levels, which supported cerebral blood flow and kept the patient awake [55]. This approach aligns with findings from Li and colleagues, who noted accentuated hyperoxygenation and normal PaCO2 in patients undergoing AAA and MAC, attributing the absence of respiratory complications to lower remifentanil and higher dexmedetomidine use in both groups [60].

The iECoG protocol used in our series is consistent with configurations reported for awake, mapping-guided epilepsy surgeries, where cortical stimulation mapping is combined with simultaneous iECoG to detect after-discharges and delineate functional and electrophysiological resection boundaries [52, 61, 62]. However, iECoG is not mandatory across all awake epilepsy series. For example, Uda et al. used awake craniotomy primarily to enable functional mapping in selected diagnostic scenarios, and iECoG was not applied systematically; in those cases, stereoelectroencephalography had already provided sufficient electrophysiological localization [63]. In our workflow, iECoG served mainly as a confirmatory and tailored tool, with negative post-resection iECoG verifying electrical completeness rather than prompting enlargement of the planned CAH, a process also guided by IONM and neurophysiological testing.

Intraoperative seizures are documented complications during AC for gliomas, with incidence rates ranging from 3% to 30% [64]. Studies suggest that combining levetiracetam with perampanel significantly reduces seizure frequency compared to levetiracetam monotherapy, likely through plasma stabilization and modulation of tumor-related glutamate activity [65]. We observed no intraoperative seizures, potentially due to established antiseizure drug regimens. We acknowledge that the perioperative use of perampanel may not be clinically justified in patients with ongoing antiseizure medication, given its delayed pharmacokinetics and cost [66–68].

The only significant difference observed between the groups was the duration of surgery, which was longer in the AAA group compared to the GA group. As highlighted in previous research, prolonged surgical durations are associated with an increased risk of complications, including surgical site infections (SSI). For instance, a systematic review and meta-analysis by Cheng et al. demonstrated that complications, including infections and cardiovascular issues, were more frequent as surgical time increased [69]. Similarly, Kaye and collaborator found a direct correlation between longer operative durations and higher rates of SSI in neurosurgical procedures [70]. In our case, the infection observed in the AAA group may have been a consequence of the prolonged surgery time.

Regarding the surgery, the standard basal CAH approach [41] was planned to be adapted only when the EZ extended toward eloquent cortex. Intraoperative language mapping in all 11 AAA patients confirmed that eloquent areas were distant from the EZ, allowing planned resections to proceed without modification and serving primarily as a confirmatory measure (Table 4). In the GA group, all resections adhered to preoperative CAH margins without intraoperative changes.

Further intraoperative neurophysiological monitoring could be also implemented in the GA arm, such as somatosensory and motor evoked potentials [61, 62], modalities that our team has used in prior work [43]. However, we did not apply these techniques in the GA group because the epileptogenic zone was clearly confined to mesial temporal structures, and no eloquent cortical areas were at risk of resection. Nevertheless, preoperative tractography was systematically reviewed for all patients in both groups as part of our neuroimaging protocol, allowing detailed assessment of major white matter pathways, including the arcuate fasciculus. In all cases, this bundle was located outside the surgical corridor and remained unaffected by the mesial resection corroborated in postsurgical imaging.

### Postsurgical outcomes of the patients based on the anesthesia modality

Both the duration of hospitalization and complication rates in our cohort (Table 5) indicate good efficacy when compared to other studies [11, 37, 51, 55, 71]. Prolonged hospital stays can predispose patients to complications unrelated to surgery. In our case, both groups had hospital stays ranging from 5.3 to 7.3 days. Brown and colleagues highlighted that AAA reduces complication rates associated with GA, presenting a clear advantage over other procedures [11]. Similarly, Mofatteh et al. described AAA as a safe procedure with fewer postoperative complications, reporting mostly non-significant findings across studies in their systematic review [9]. We reported in both groups low complication rates. In the GA group, there were no complications, whereas in the AAA group, one patient had an SSI, perhaps due to the prolonged surgical time, which did not lead to long-term issues (Table 5).

Several advantages of AAA have been reported in tumor surgery, especially for gliomas located near or within eloquent areas of the cortex. AAA reduces the risk of neurological deficits—such as motor, sensory, language, and visual impairments—and improves safety by lowering postoperative complications [9, 51, 61, 72]. In Kim et al.‘s study of 309 patients, the incidence of permanent neurological deficits was 7% in AAA cases [51]. In contrast, no permanent neurological deficits were observed in our series under AAA (Table 5).

Concerning language impairment, naming difficulties occur in 14 to 60% of patients undergoing epilepsy surgery with AAA, even when seizure control is achieved after surgery [61]. In our series of patients managed with the AAA protocol, none of these deficits were observed. On visual field defects, two patients in the GA group developed homonymous quadrantanopia within 12 months; one case was transient and the other permanent at follow-up. Of note, these deficits occurred exclusively in GA group, where the resection was confined to the presurgical plan, although the events do not appear to be specifically related to the type of anesthetic modality. Schmeiser et al. reported a 73% incidence of visual field deficits in a cohort of 276 patients who underwent epilepsy surgery for DRE secondary to temporal lobe epilepsy [73]. Injury to Meyer’s loop, particularly the geniculocalcarine fibers, is a common complication during these procedures and often results in contralateral homonymous quadrantanopia [74]. Although visual field were assessed pre- and postoperatively using standard automated perimetry, intraoperative monitoring of the visual pathway could be incorporated into the protocol in future studies to clarify any visual field deficits that develop after surgery.

In the context of AAA protocol in epilepsy surgery, only three prior studies have been documented. The first, by Minkin et al., analyzed 97 patients with DRE due to focal cortical dysplasia (FCD) in eloquent areas, divided into three groups based on the anesthetic and surgical approach. The AAA group, comprising 14 patients, showed better seizure control compared to those under GA, with 71% achieving ILAE class I. Language deficits occurred in 21% of the AAA group, a rate not significantly different from that in the GA group [61]. Vigren et al. reported four cases of epilepsy surgery using AAA in patients with FCD located in various cortical regions. All achieved Engel class I outcomes without postoperative neurological deficits [62]. A study by Guibourd de Luzinais et al., involving a broader spectrum of epileptogenic pathologies, evaluated AAA with mapping-guided resections in 22 patients with lesions adjacent to eloquent brain areas, including 10 patients with no clearly identifiable lesion on imaging. The study reported that 72.7% of patients achieved Engel class I outcomes, and none developed permanent neurological impairments [52]. In our series of patients with MTLE-HS under AAA, seizure outcomes and neurological preservation were consistent with prior reports [52, 61, 62], with 55% of patients achieving ILAE class I and 18% class II, without observed language deficits (Table 5).

### Limitations of the study

One key limitation of this study was the small number of patients eligible for both epilepsy surgery and the AAA modality. This limited pool reduced the number of patients who underwent surgery based on neuroimaging findings and clinical features. Additionally, only one subset of these candidates was selected for the AAA technique, as they needed to tolerate being awake and cooperate during the neurological examination, while also not having comorbidities that could compromise the patient’s life during the anesthesia.

Aside from the surgical duration, the study did not show significant differences between AAA and GA regarding intraoperative results and postoperative outcomes. This lack of difference is likely due to the small sample size, which limited the power of the study to detect meaningful variations between the two anesthesia modalities.

Although the retrospective design and sample size preclude a definitive assessment of superiority, this study aims to provide preliminary evidence supporting the feasibility and non-inferiority of AAA in selected patients, serving as a foundation for future prospective research. To fully establish the efficacy of AAA, a larger prospective study with longer follow-up is needed to better assess its potential benefits.

## Conclusion

In this retrospective case-control study comparing AAA and GA modalities for epilepsy surgery, the results indicate that both anesthetic modalities yield similar outcomes regarding intraoperative and postoperative metrics. The analysis of presurgical characteristics, intraoperative parameters, and postoperative results did not reveal significant differences between the two groups, except for the surgery duration.

AAA may offer outcomes comparable to GA in epilepsy surgery, but due to limited evidence and longer procedure times, its widespread adoption cannot yet be recommended. Larger, multicenter studies and ongoing follow-up are needed to confirm its safety and efficacy in temporal lobe resections involving eloquent areas.

The AAA technique is particularly valuable in dominant-hemisphere MTLE-HS resections because it enables real-time assessment of language functions during manipulation of regions closely connected to both the mesial limbic system and the temporal neocortex. While CAH is traditionally performed under GA, AAA offers an important advantage in clinical situations where the epileptogenic zone may extend slightly beyond strictly mesial structures or where its lateral boundaries are less sharply defined preoperatively. In these cases, even minimal unintended extension of the resection could affect language or cognitive outcomes. By allowing continuous functional monitoring, AAA helps ensure the preservation of eloquent functions and supports optimal postoperative quality of life.

## Data Availability

No datasets were generated or analysed during the current study.

## References

[1] Zhang YJ, Kong XM, Lv JJ, Yang CH, Li XY, Yang XT, Guo ZL, Cheng ZH (2023) Analysis of the global burden of disease study highlights the global, regional, and National trends of idiopathic epilepsy epidemiology from 1990 to 2019. Prev Med Rep 36:102522. 10.1016/j.pmedr.2023.102522 38116287 10.1016/j.pmedr.2023.102522 PMC10728447

[2] French JA (2007) Refractory epilepsy: clinical overview. Epilepsia 48(Suppl 1):3–7. 10.1111/j.1528-1167.2007.00992.x 17316406 10.1111/j.1528-1167.2007.00992.x

[3] Engel J (2018) Jr. The current place of epilepsy surgery. Curr Opin Neurol 31:192–197. 10.1097/WCO.0000000000000528 29278548 10.1097/WCO.0000000000000528 PMC6009838

[4] Noe K, Sulc V, Wong-Kisiel L, Wirrell E, Van Gompel JJ, Wetjen N, Britton J, So E, Cascino GD, Marsh WR et al (2013) Long-term outcomes after nonlesional extratemporal lobe epilepsy surgery. JAMA Neurol 70:1003–1008. 10.1001/jamaneurol.2013.209 23732844 10.1001/jamaneurol.2013.209 PMC3920594

[5] Bindra A, Chouhan RS, Prabhakar H, Chandra PS, Tripathi M (2015) Perioperative anesthetic implications of epilepsy surgery: a retrospective analysis. J Anesth 29:229–234. 10.1007/s00540-014-1919-2 25288505 10.1007/s00540-014-1919-2

[6] Larkin CM, O’Brien DF, Maheshwari D (2019) Anaesthesia for epilepsy surgery. BJA Educ 19:383–389. 10.1016/j.bjae.2019.08.001 33456862 10.1016/j.bjae.2019.08.001 PMC7807957

[7] Shetty A, Pardeshi S, Shah VM, Kulkarni A (2016) Anesthesia considerations in epilepsy surgery. Int J Surg 36:454–459. 10.1016/j.ijsu.2015.07.006 26188082 10.1016/j.ijsu.2015.07.006

[8] Al Fudhaili AN, Al-Busaidi F, Madan ZM, Al Issa MS, Al Mamria MH, Al-Saadi T (2023) Awake craniotomy surgery in pediatrics: A systematic review. World Neurosurg 179:82–87. 10.1016/j.wneu.2023.08.040 37595837 10.1016/j.wneu.2023.08.040

[9] Mofatteh M, Mashayekhi MS, Arfaie S, Wei H, Kazerouni A, Skandalakis GP, Pour-Rashidi A, Baiad A, Elkaim L, Lam J et al (2023) Awake craniotomy during pregnancy: A systematic review of the published literature. Neurosurg Rev 46:290. 10.1007/s10143-023-02187-x 37910275 10.1007/s10143-023-02187-x PMC10620271

[10] Sattari SA, Rincon-Torroella J, Sattari AR, Feghali J, Yang W, Kim JE, Xu R, Jackson CM, Mukherjee D, Lin SC et al (2024) Awake versus asleep craniotomy for patients with eloquent glioma: A systematic review and Meta-Analysis. Neurosurgery 94:38–52. 10.1227/neu.0000000000002612 37489887 10.1227/neu.0000000000002612

[11] Brown T, Shah AH, Bregy A, Shah NH, Thambuswamy M, Barbarite E, Fuhrman T, Komotar RJ (2013) Awake craniotomy for brain tumor resection: the rule rather than the exception? J Neurosurg Anesthesiol 25:240–247. 10.1097/ANA.0b013e318290c230 23603885 10.1097/ANA.0b013e318290c230

[12] Groshev A, Padalia D, Patel S, Garcia-Getting R, Sahebjam S, Forsyth PA, Vrionis FD, Etame AB (2017) Clinical outcomes from maximum-safe resection of primary and metastatic brain tumors using awake craniotomy. Clin Neurol Neurosurg 157:25–30. 10.1016/j.clineuro.2017.03.017 28384595 10.1016/j.clineuro.2017.03.017

[13] Manninen PH, Tan TK (2002) Postoperative nausea and vomiting after craniotomy for tumor surgery: a comparison between awake craniotomy and general anesthesia. J Clin Anesth 14:279–283. 10.1016/s0952-8180(02)00354-9 12088812 10.1016/s0952-8180(02)00354-9

[14] Bonhomme V, Franssen C, Hans P (2009) Awake craniotomy. Eur J Anaesthesiol 26:906–912. 10.1097/EJA.0b013e32833000c5 19617839 10.1097/EJA.0b013e32833000c5

[15] Kulikov A, Lubnin A (2018) Anesthesia for awake craniotomy. Curr Opin Anaesthesiol 31:506–510. 10.1097/ACO.0000000000000625 29994938 10.1097/ACO.0000000000000625

[16] Natalini D, Ganau M, Rosenkranz R, Petrinic T, Fitzgibbon K, Antonelli M, Prisco L (2022) Comparison of the Asleep-Awake-Asleep technique and monitored anesthesia care during awake craniotomy: A systematic review and Meta-analysis. J Neurosurg Anesthesiol 34:e1–e13. 10.1097/ANA.0000000000000675 31972627 10.1097/ANA.0000000000000675

[17] Blumcke I, Thom M, Aronica E, Armstrong DD, Bartolomei F, Bernasconi A, Bernasconi N, Bien CG, Cendes F, Coras R et al (2013) International consensus classification of hippocampal sclerosis in Temporal lobe epilepsy: a task force report from the ILAE commission on diagnostic methods. Epilepsia 54:1315–1329. 10.1111/epi.12220 23692496 10.1111/epi.12220

[18] Kiernan RJ, Mueller J, Langston JW, Van Dyke C (1987) The neurobehavioral cognitive status examination: a brief but quantitative approach to cognitive assessment. Ann Intern Med 107:481–485. 10.7326/0003-4819-107-4-481 3631786 10.7326/0003-4819-107-4-481

[19] Shin MS, Park SY, Park SR, Seol SH, Kwon JS (2006) Clinical and empirical applications of the Rey-Osterrieth complex figure test. Nat Protoc 1:892–899. 10.1038/nprot.2006.115 17406322 10.1038/nprot.2006.115

[20] Ryan JJ, Geisser ME (1986) Validity and diagnostic accuracy of an alternate form of the Rey auditory verbal learning test. Arch Clin Neuropsychol 1:209–217 14591149

[21] Oldfield RC (1971) The assessment and analysis of handedness: the Edinburgh inventory. Neuropsychologia 9:97–113. 10.1016/0028-3932(71)90067-4 5146491 10.1016/0028-3932(71)90067-4

[22] Trejo-Martínez D, Manjarrez-Garduño DA, Becerril-Montes H, Granados-Domínguez L, Velasco-Monroy AL (2018) Language lateralisation through dichotic listening in a group of patients with temporal lobe epilepsy. Revista Médica del Hospital General de México 81:190–196. 10.1016/j.hgmx.2017.05.006

[23] Fong MWM, Van Patten R, Fucetola RP (2019) The factor structure of the Boston diagnostic aphasia Examination, third edition. J Int Neuropsychol Soc 25:772–776. 10.1017/S1355617719000237 31030708 10.1017/S1355617719000237

[24] Janssens M, Hartstein G (2001) Management of difficult intubation. Eur J Anaesthesiol 18:3–12. 10.1046/j.0265-0215.2000.00777.x 11270007 10.1046/j.0265-0215.2000.00777.x

[25] Meng L, McDonagh DL, Berger MS, Gelb AW (2017) Anesthesia for awake craniotomy: a how-to guide for the occasional practitioner. Can J Anaesth 64:517–529. 10.1007/s12630-017-0840-1 28181184 10.1007/s12630-017-0840-1

[26] Kim SH, Choi SH (2020) Anesthetic considerations for awake craniotomy. Anesth Pain Med (Seoul) 15:269–274. 10.17085/apm.20050 33329824 10.17085/apm.20050 PMC7713838

[27] D’Onofrio G, Izzi A, Manuali A, Bisceglia G, Tancredi A, Marchello V, Recchia A, Tonti MP, Icolaro N, Fazzari E et al (2023) Anesthetic management for awake craniotomy applied to neurosurgery. Brain Sci 13. 10.3390/brainsci13071031 10.3390/brainsci13071031 PMC10377309 37508963

[28] Sokhal N, Rath GP, Chaturvedi A, Dash HH, Bithal PK, Chandra PS (2015) Anaesthesia for awake craniotomy: a retrospective study of 54 cases. Indian J Anaesth 59:300–305. 10.4103/0019-5049.156878 26019355 10.4103/0019-5049.156878 PMC4445152

[29] Osborn I, Sebeo J (2010) Scalp block during craniotomy: a classic technique revisited. J Neurosurg Anesthesiol 22:187–194. 10.1097/ANA.0b013e3181d48846 20479675 10.1097/ANA.0b013e3181d48846

[30] Al Mashani AM, Ali A, Chatterjee N, Suri N, Das S (2018) Awake craniotomy during pregnancy. J Neurosurg Anesthesiol 30:372–373. 10.1097/ANA.0000000000000424 28240617 10.1097/ANA.0000000000000424

[31] Gaudray E, C NG, Martin E, Lyochon A, Dagain A, Bordes J, Cordier PY, Lacroix G (2020) Efficacy of scalp nerve blocks using ropivacaine 0,75% associated with intravenous dexamethasone for postoperative pain relief in craniotomies. Clin Neurol Neurosurg 197:106125. 10.1016/j.clineuro.2020.106125 32836063 10.1016/j.clineuro.2020.106125

[32] Yang Y, Ou M, Zhou H, Tan L, Hu Y, Li Y, Zhu T (2020) Effect of scalp nerve block with ropivacaine on postoperative pain in patients undergoing craniotomy: A Randomized, double blinded study. Sci Rep 10:2529. 10.1038/s41598-020-59370-z 32054899 10.1038/s41598-020-59370-z PMC7018808

[33] Frerk C, Mitchell VS, McNarry AF, Mendonca C, Bhagrath R, Patel A, O’Sullivan EP, Woodall NM, Ahmad I (2015) Difficult airway society intubation guidelines working, g. Difficult airway society 2015 guidelines for management of unanticipated difficult intubation in adults. Br J Anaesth 115:827–848. 10.1093/bja/aev371 26556848 10.1093/bja/aev371 PMC4650961

[34] Lobo FA, Wagemakers M, Absalom AR (2016) Anaesthesia for awake craniotomy. Br J Anaesth 116:740–744. 10.1093/bja/aew113 27199306 10.1093/bja/aew113

[35] Eleveld DJ, Proost JH, Cortinez LI, Absalom AR, Struys MM (2014) A general purpose Pharmacokinetic model for Propofol. Anesth Analg 118:1221–1237. 10.1213/ANE.0000000000000165 24722258 10.1213/ANE.0000000000000165

[36] Hannivoort LN, Eleveld DJ, Proost JH, Reyntjens KM, Absalom AR, Vereecke HE, Struys MM (2015) Development of an optimized Pharmacokinetic model of Dexmedetomidine using Target-controlled infusion in healthy volunteers. Anesthesiology 123:357–367. 10.1097/ALN.0000000000000740 26068206 10.1097/ALN.0000000000000740

[37] Dilmen OK, Akcil EF, Oguz A, Vehid H, Tunali Y (2017) Comparison of conscious sedation and Asleep-Awake-Asleep techniques for awake craniotomy. J Clin Neurosci 35:30–34. 10.1016/j.jocn.2016.10.007 27771234 10.1016/j.jocn.2016.10.007

[38] Brown EN, Pavone KJ, Naranjo M (2018) Multimodal general anesthesia: theory and practice. Anesth Analg 127:1246–1258. 10.1213/ANE.0000000000003668 30252709 10.1213/ANE.0000000000003668 PMC6203428

[39] Young JS, Lee AT, Chang EF (2021) A review of cortical and subcortical stimulation mapping for Language. Neurosurgery 89:331–342. 10.1093/neuros/nyaa436 33444451 10.1093/neuros/nyaa436 PMC8492609

[40] Al-Adli NN, Young JS, Sibih YE, Berger MS (2023) Technical aspects of motor and Language mapping in glioma patients. Cancers (Basel) 15. 10.3390/cancers15072173 10.3390/cancers15072173 PMC10093517 37046834

[41] Alonso Vanegas MA, Lew SM, Morino M, Sarmento SA (2017) Microsurgical techniques in Temporal lobe epilepsy. Epilepsia 58(Suppl 1):10–18. 10.1111/epi.13684 28386927 10.1111/epi.13684

[42] San-juan D, Tapia CA, Gonzalez-Aragon MF, Martinez Mayorga A, Staba RJ, Alonso-Vanegas M (2011) The prognostic role of electrocorticography in tailored Temporal lobe surgery. Seizure 20:564–569. 10.1016/j.seizure.2011.04.006 21616682 10.1016/j.seizure.2011.04.006

[43] Sandoval-Bonilla BA, Luna IV, Arritola-Uriarte A, San-Juan D, Garcia-Iturbide R, Gress Mendoza AE, Pulido LC, Gonzalez LH, Bahena AL, De la Vargas C (2025) Intraoperative monitoring during awake craniotomy for glioblastoma resection in the second trimester of pregnancy. A case report and literature review. Clin Neurophysiol Pract 10:63–69. 10.1016/j.cnp.2025.02.006 40124178 10.1016/j.cnp.2025.02.006 PMC11930422

[44] San-Juan D, Alonso-Vanegas MA, Trenado C, Hernandez-Segura N, Espinoza-Lopez DA, Gonzalez-Perez B, Cobos-Alfaro E, Zuniga-Gazcon H, de Fernandez-Gonzalez MDC, Hernandez-Ruiz A (2017) Electrocorticographic patterns in epilepsy surgery and Long-Term outcome. J Clin Neurophysiol 34:520–526. 10.1097/WNP.0000000000000407 28786834 10.1097/WNP.0000000000000407

[45] Mathon B, Bedos Ulvin L, Adam C, Baulac M, Dupont S, Navarro V, Cornu P, Clemenceau S (2015) Surgical treatment for mesial Temporal lobe epilepsy associated with hippocampal sclerosis. Rev Neurol (Paris) 171:315–325. 10.1016/j.neurol.2015.01.561 25746582 10.1016/j.neurol.2015.01.561

[46] Zijlmans M, Zweiphenning W, van Klink N (2019) Changing concepts in presurgical assessment for epilepsy surgery. Nat Rev Neurol 15:594–606. 10.1038/s41582-019-0224-y 31341275 10.1038/s41582-019-0224-y

[47] Marin-Romero B, Tirapu-Ustarroz J, Chiofalo MF (2020) [Neuropsychological assessment protocol for adults in epilepsy surgery]. Rev Neurol 70:341–347. 10.33588/rn.7009.2019441 32329047 10.33588/rn.7009.2019441

[48] Qadri S, Dave H, Das R, Alick-Lindstrom S (2021) Beyond the wada: an updated approach to pre-surgical Language and memory testing: an updated review of available evaluation techniques and recommended workflow to limit Wada test use to essential clinical cases. Epilepsy Res 174:106673. 10.1016/j.eplepsyres.2021.106673 34082393 10.1016/j.eplepsyres.2021.106673

[49] Hund-Georgiadis M, Lex U, Friederici AD, von Cramon DY (2002) Non-invasive regime for Language lateralization in right- and left-handers by means of functional MRI and dichotic listening. Exp Brain Res 145:166–176. 10.1007/s00221-002-1090-0 12110956 10.1007/s00221-002-1090-0

[50] Fernandes MA, Smith ML, Logan W, Crawley A, McAndrews MP (2006) Comparing language lateralization determined by dichotic listening and fMRI activation in frontal and Temporal lobes in children with epilepsy. Brain Lang 96:106–114. 10.1016/j.bandl.2005.06.006 16083954 10.1016/j.bandl.2005.06.006

[51] Kim SS, McCutcheon IE, Suki D, Weinberg JS, Sawaya R, Lang FF, Ferson D, Heimberger AB, DeMonte F, Prabhu SS (2009) Awake craniotomy for brain tumors near eloquent cortex: correlation of intraoperative cortical mapping with neurological outcomes in 309 consecutive patients. Neurosurgery 64:836–845. 10.1227/01.NEU.0000342405.80881.81. discussion 345–836 19404147 10.1227/01.NEU.0000342405.80881.81

[52] Guibourd de Luzinais M, Engelhardt J, Ollivier M, Planchon C, Gallice T, Michel V, de Montaudouin M, Aupy J, Penchet G (2024) Awake surgery with mapping-based resection to treat focal epilepsy in eloquent brain areas. Acta Neurochir (Wien) 166:430. 10.1007/s00701-024-06326-1 39472357 10.1007/s00701-024-06326-1

[53] Hermann BP, Wyler AR (1988) Effects of anterior Temporal lobectomy on Language function: a controlled study. Ann Neurol 23:585–588. 10.1002/ana.410230610 3408239 10.1002/ana.410230610

[54] Ozlu O (2018) Anaesthesiologist’s approach to awake craniotomy. Turk J Anaesthesiol Reanim 46:250–256. 10.5152/TJAR.2018.56255 30140530 10.5152/TJAR.2018.56255 PMC6101712

[55] Rajan S, Cata JP, Nada E, Weil R, Pal R, Avitsian R (2013) Asleep-awake-asleep craniotomy: a comparison with general anesthesia for resection of supratentorial tumors. J Clin Neurosci 20:1068–1073. 10.1016/j.jocn.2012.09.031 23453156 10.1016/j.jocn.2012.09.031

[56] Akavipat P, Sookplung P, Lekprasert V, Kasemsiri C, Lerdsirisophon S (2024) Dexmedetomidine for awake craniotomy: systematic review and meta-analysis. J Clin Neurosci 127:110765. 10.1016/j.jocn.2024.110765 39079421 10.1016/j.jocn.2024.110765

[57] Weenink RP, de Jonge SW, van Hulst RA, Wingelaar TT, van Ooij PAM, Immink RV, Preckel B, Hollmann MW (2020) Perioperative hyperoxyphobia: justified or not? Benefits and harms of hyperoxia during surgery. J Clin Med 9. 10.3390/jcm9030642 10.3390/jcm9030642 PMC7141263 32121051

[58] Reber A, Engberg G, Wegenius G, Hedenstierna G (1996) Lung aeration. The effect of pre-oxygenation and hyperoxygenation during total intravenous anaesthesia. Anaesthesia 51:733–737. 10.1111/j.1365-2044.1996.tb07885.x 8795314 10.1111/j.1365-2044.1996.tb07885.x

[59] Horncastle E, Lumb AB (2019) Hyperoxia in anaesthesia and intensive care. BJA Educ 19:176–182. 10.1016/j.bjae.2019.02.005 33456888 10.1016/j.bjae.2019.02.005 PMC7807946

[60] Li SZ, Su N, Wu S, Fei XW, He X, Zhang JX, Wang XH, Zhang HP, Bai XG, Cheng G et al (2022) Monitored anesthesia care and asleep-awake-asleep techniques combined with multiple monitoring for resection of gliomas in eloquent brain areas: a retrospective analysis of 225 patients. Chin Neurosurg J 8. 10.1186/s41016-022-00311-2 10.1186/s41016-022-00311-2 PMC9801549 36582003

[61] Minkin K, Gabrovski K, Karazapryanov P, Milenova Y, Sirakov S, Karakostov V, Romanski K, Dimova P (2021) Awake epilepsy surgery in patients with focal cortical dysplasia. World Neurosurg 151:e257–e264. 10.1016/j.wneu.2021.04.021 33872840 10.1016/j.wneu.2021.04.021

[62] Vigren P, Eriksson M, Duffau H, Wretman A, Lindehammar H, Milos P, Richter J, Karlsson T, Gauffin H (2020) Experiences of awake surgery in non-tumoural epilepsy in eloquent localizations. Clin Neurol Neurosurg 199:106251. 10.1016/j.clineuro.2020.106251 33031989 10.1016/j.clineuro.2020.106251

[63] Uda T, Tanoue Y, Kawashima T, Yindeedej V, Nishijima S, Kunihiro N, Umaba R, Ishimoto K, Goto T (2024) Awake craniotomy in epilepsy surgery: a case series and proposal for three different scenarios. Brain Sci 14. 10.3390/brainsci14100958 10.3390/brainsci14100958 PMC11506450 39451973

[64] Paquin-Lanthier G, Subramaniam S, Leong KW, Daniels A, Singh K, Takami H, Chowdhury T, Bernstein M, Venkatraghavan L (2023) Risk factors and characteristics of intraoperative seizures during awake craniotomy: A retrospective cohort study of 562 consecutive patients with a Space-occupying brain lesion. J Neurosurg Anesthesiol 35:194–200. 10.1097/ANA.0000000000000798 34411059 10.1097/ANA.0000000000000798

[65] Motomura K, Chalise L, Shimizu H, Yamaguchi J, Nishikawa T, Ohka F, Aoki K, Tanahashi K, Hirano M, Wakabayashi T et al (2021) Intraoperative seizure outcome of Levetiracetam combined with perampanel therapy in patients with glioma undergoing awake brain surgery. J Neurosurg 135:998–1007. 10.3171/2020.8.JNS201400 33482638 10.3171/2020.8.JNS201400

[66] Greenwood J, Valdes J (2016) Perampanel (Fycompa): A review of clinical efficacy and safety in epilepsy. P T 41:683–698 27904300 PMC5083075

[67] Besag FM, Patsalos PN (2016) Clinical efficacy of perampanel for partial-onset and primary generalized tonic-clonic seizures. Neuropsychiatr Dis Treat 12:1215–1220. 10.2147/NDT.S83842 27274257 10.2147/NDT.S83842 PMC4876101

[68] Patsalos PN (2015) The clinical Pharmacology profile of the new antiepileptic drug perampanel: a novel noncompetitive AMPA receptor antagonist. Epilepsia 56:12–27. 10.1111/epi.12865 25495693 10.1111/epi.12865

[69] Cheng H, Clymer JW, Po-Han Chen B, Sadeghirad B, Ferko NC, Cameron CG, Hinoul P (2018) Prolonged operative duration is associated with complications: a systematic review and meta-analysis. J Surg Res 229:134–144. 10.1016/j.jss.2018.03.022 29936980 10.1016/j.jss.2018.03.022

[70] Bekelis K, Coy S, Simmons N (2016) Operative duration and risk of surgical site infection in neurosurgery. World Neurosurg 94:551–555. 10.1016/j.wneu.2016.07.077 27485528 10.1016/j.wneu.2016.07.077

[71] Eseonu CI, ReFaey K, Garcia O, John A, Quinones-Hinojosa A, Tripathi P (2017) Awake craniotomy anesthesia: a comparison of the monitored anesthesia care and Asleep-Awake-Asleep techniques. World Neurosurg 104:679–686. 10.1016/j.wneu.2017.05.053 28532922 10.1016/j.wneu.2017.05.053

[72] Wolfson R, Soni N, Shah AH, Hosein K, Sastry A, Bregy A, Komotar RJ (2015) The role of awake craniotomy in reducing intraoperative visual field deficits during tumor surgery. Asian J Neurosurg 10:139–144. 10.4103/1793-5482.161189 26396597 10.4103/1793-5482.161189 PMC4553722

[73] Schmeiser B, Daniel M, Kogias E, Bohringer D, Egger K, Yang S, Foit NA, Schulze-Bonhage A, Steinhoff BJ, Zentner J et al (2017) Visual field defects following different resective procedures for mesiotemporal lobe epilepsy. Epilepsy Behav 76:39–45. 10.1016/j.yebeh.2017.08.037 28954709 10.1016/j.yebeh.2017.08.037

[74] Powell HW, Parker GJ, Alexander DC, Symms MR, Boulby PA, Wheeler-Kingshott CA, Barker GJ, Koepp MJ, Duncan JS (2005) MR tractography predicts visual field defects following Temporal lobe resection. Neurology 65:596–599. 10.1212/01.wnl.0000172858.20354.73 16116123 10.1212/01.wnl.0000172858.20354.73

